# Polymorphisms in the *ADRB2 *gene and Graves disease: a case-control study and a meta-analysis of available evidence

**DOI:** 10.1186/1471-2350-10-26

**Published:** 2009-03-13

**Authors:** Xun Chu, Yan Dong, Min Shen, Lingling Sun, Changzheng Dong, Yi Wang, Beilan Wang, Kaiyue Zhang, Qi Hua, Shijie Xu, Wei Huang

**Affiliations:** 1 Ruijin Hospital, School of Medicine, Shanghai Jiaotong University, Shanghai, PR China; 2 Department of Genetics, Shanghai-MOST Key Laboratory of Health and Disease Genomics, Chinese National Human Genome Center, Shanghai, PR China; 3 Department of Endocrinology, Xinhua Hospital, School of Medicine, Shanghai Jiaotong University, Shanghai, PR China

## Abstract

**Background:**

The beta-2-Adrenergic receptor (*ADRB2*) gene on chromosome 5q33.1 is an important immunoregulatory factor. We and others have previously implicated chromosomal region 5q31-33 for contribution to the genetic susceptibility to Graves disease (GD) in East-Asian populations. Two recent studies showed associations between the single nucleotide polymorphism (SNP) rs1042714 in the *ADRB2 *gene and GD. In this study, we aimed to fully investigate whether the *ADRB2 *gene conferred susceptibility to GD in Chinese population, and to perform a meta-analysis of association between *ADRB2 *and GD.

**Methods:**

Approximately 1 kb upstream the transcription start site and the entire coding regions of the *ADRB2 *gene were resequenced in 48 Han Chinese individuals to determine the linkage disequilibrium (LD) patterns. Tag SNPs were selected and genotyped in a case-control collection of 1,118 South Han Chinese subjects, which included 428 GD patients and 690 control subjects. A meta-analysis was performed with the data obtained in the present samples and those available from prior studies.

**Results:**

Fifteen SNPs in the *ADRB2 *gene were identified by resequencing and one SNP was novel. Ten tag SNPs were investigated further to assess association of *ADRB2 *in the case-control collection. Neither individual tag SNP nor haplotypes showed association with GD in Han Chinese population (P > 0.05). Our meta-analysis of the *ADRB2 *SNP rs1042714 measured heterogeneity between the ethnic groups (I^2 ^= 53.1%) and no association to GD was observed in the overall three studies with a random effects model (OR = 1.13, 95% CI, 0.95 to 1.36; P = 0.18). However, significant association was found from the combined data of Caucasian population with a fixed effects model (OR = 1.18, 95% CI, 1.06 to 1.32; P = 0.002; I^2 ^= 5.9%).

**Conclusion:**

Our study indicated that the *ADRB2 *gene did not exert a substantial influence on GD susceptibility in Han Chinese population, but contributed to a detectable GD risk in Caucasian population. This inconsistency resulted largely from between-ethnicity heterogeneity.

## Background

Graves disease (GD) affects 0.5–1% of the general population[1], and results from the presence of autoantibodies to the thyroid-stimulating hormone receptor (*TSHR*), leading to over-activity of the thyroid gland. Genome-wide screens conducted in GD have identified a number of regions of linkage, although independent replication and novel candidate loci are to be awaited. Our previous study[2], along with others[3,4], has suggested that chromosomal region 5q31-33 may contain a locus that contributes to the genetic susceptibility to GD in Eastern-Asian populations. *ADRB2*, a 1242-base pair intronless gene, is located on the long arm of chromosome 5q33.1. At least thirteen SNPs have been identified in the gene region[5,6]. Two activity-related polymorphisms (rs1042713 and rs1042714) with high allelic frequency in the general population are single nucleic acid substitutions at positions 46 (A-G) and 79 (C-G), corresponding to substitutions of glycine for arginine at amino acid position 16 and glutamate for glutamine at amino acid position 27[5,6].

Recently, these two SNPs (rs1042713 and rs1042714) and another SNP located at position -367 (rs11959427) were reported association with GD in a Polish case-control collection[7]. The result indicated that Gln27 carriers (rs1042714CC or rs1042714GC genotypes) had increased risk of GD. Additionally, the genome-wide association study (GWAS) of the Wellcome Trust Case Control Consortium (WTCCC) *et al *also showed positive signal in the *ADRB2 *gene[8]. Although three SNPs (rs12654778, rs1042713 and rs1042714) in the *ADRB2 *gene genotyped in the study of WTCCC *et al. *did not meet a point-wise significance level of P < 10^-3 ^for the Cochran-Armitage test for trend, the original analyses showed significant association of rs1042713 and rs1042714 to GD (P = 0.04 for rs1042713 and P = 0.02 for rs1042714).

ADRB2 is a G protein coupled receptor that is expressed by all lymphoid cells, with the exception of T helper (Th) 2 cells[9]. Norepinephrine and epinephrine, through stimulation of the ADRB2-cAMP-protein kinase A pathway, cause a selective suppression of Th1 responses and cellular immunity, and a Th2 shift toward dominance of humoral immunity[9]. Moreover, various polymorphisms in the *ADRB2 *gene were demonstrated as leading to a variation of ADRB2 expression on the cell surface[10,11]. Therefore, the polymorphisms may influence the activity of ADRB2, and disturb the Th1/Th2 cytokine balance, which is an important factor in the pathogenesis of GD. In addition, *ADRB2 *had been reported association with another autoimmune disease, rheumatoid arthritis [12]. This increased the possibility of *ADRB2 *association with GD, as different autoimmune diseases may share similar genetic etiologies. Given its chromosomal location within a region of genetic linkage to GD and the role of ADRB2 on the immune response, the *ADRB2 *gene makes a plausible candidate for GD and autoimmunity in general.

In the present study, we set to determine whether *ADRB2 *was a susceptibility locus for GD in Han Chinese population and conduct a global meta-analysis of the *ADRB2 *variation association with GD. Firstly, we identified the variants of *ADRB2 *by resequencing the 5' flanking region and the entire coding region; then we defined linkage disequilibrium (LD) among SNPs across the *ADRB2 *genomic interval, and selected tag SNPs. Secondly, we conducted a case-control association study (428 GD cases and 690 controls) using the tag SNPs, which included three aforementioned polymorphisms. Finally, we performed a meta-analysis combining the result of our present study and the data sets of the two previously published studies.

## Methods

### Subjects

Unrelated southern Han Chinese patients with GD were recruited from hospitals in Shanghai. The diagnosis of GD was based on documented clinical and biochemical evidence of hyperthyroidism, diffused goiter, and the presence of at least one of the following: positive TSH receptor antibody tests, diffusely increased 131I (iodine-131) uptake in the thyroid gland, or presence of exophthalmos. The severity of Graves' ophthalmopathy (GO) was assessed according to the NOSPECS classification[13]. Information was also collected from examination of medical records and all other available sources. All control subjects were given tests for thyroid function and autoantibody status, and anyone showing evidence of subclinical autoimmune thyroid disease (AITD) was removed from the study. Control subjects with known family history of autoimmune disease were also excluded.

In total, 428 GD patients and 690 unrelated control subjects were recruited for the case-control study. Of the GD patients, 132 showed ophthalmopathy defined as ATA class III or worse and were classified as GO (+). A sample set of 24 GD cases and 24 controls was used for resequencing. With informed consent, blood samples were collected from all the participants for DNA preparation as well as biochemical measurements. Genomic DNA was isolated from peripheral blood leukocytes with FlexiGene DNA Kit (Qiagenn Hilden, Germany). This project was approved by the Ethical Committee of the Chinese National Human Genome Center at Shanghai for the involvement of human subjects.

### SNP identification by resequencing

We carried out SNP identification by resequencing a 1050 bp stretch located 5'-upstream of transcription start codon and the entire coding regions of *ADRB2 *in 24 patients and 24 control subjects. The sequencing reactions were performed using Applied Biosystems BigDye (version 3.1) chemistry (Applied Biosystem, Foster City, CA, USA), and the sequences were resolved using an ABI 3730 Genetic Analyzer (Applied Biosystem, Foster City, CA, USA). Analyses of the sequence traces were performed using the Staden package[14] and were double scored by a second operator.

### Genotyping

Genotyping for the tag SNPs was performed using TaqMan chemistry (Applied Biosystem, Foster City, CA, USA). The TaqMans reaction was carried out following Applied Biosystems standard protocol. Fluorescent emissions were captured using the ABI Prisms 7900HT Sequence Detection System (Applied Biosystem, Foster City, CA, USA) and converted into genotypes using the ABI Prisms SDS 2.0 software package. The data completion rate was 98.5%.

### Statistical analysis

We used χ^2 ^analysis to evaluate the significance of differences in genotype and allele frequencies between cases and controls. Allele frequencies for cases and controls were used to calculate the odds ratio (OR) and the 95% confidence interval (95% CI). The software used for statistical calculations was the SPSS 15.0 (SPSS Inc., Chicago, IL, USA) unless specified. Genotype distributions in patients and controls were evaluated for departure from Hardy-Weinberg equilibrium by the Haploview v4.0 software[15]. From the SNPs detected by resequencing, we selected tag SNPs with a minimum threshold value of 0.8 for the r^2 ^parameter. The tag SNPs were further genotyped in the collections of 428 GD patients and 690 unrelated controls. Blocks were determined using the default method of Gabriel et al[16] and haplotypes frequencies were estimated using an implementation of the expectation maximization algorithm by Haploview v4.0. We tested association with χ^2 ^analysis for each possible combination of haplotypes derived from rs1042713 and rs1042714 using Haploview v4.0. P-values less than 0.05 were considered statistically significant.

Heterogeneity between studies was evaluated using the I^2 ^statistic for inconsistency[17] and the χ^2 ^distributed Cochran Q-statistic[18]. I^2 ^is given by 100% × (Q - df)/Q, where df denotes degrees of freedom. It describes the proportion of variation that is unlikely due to chance and is considered large for values > 50%[19]. The Q-test is the most widely used test for heterogeneity, but is recognized to have poor power when there are few studies. Hence Q is considered statistically significant for P < 0.10[17,18]. However I^2 ^is unaffected by the number of studies and consequently is useful when comparing subgroups within the overall study. The I^2 ^estimate and the Q-statistic were both computed for comparison. A fixed-effect model using Mantel-Haenszel method and a random-effects model using DerSimonian and Laird method were used to pool the results [20]. Random effects models are more conservative than fixed effects models and generate a wider confidence interval. Meta-analysis was performed using Review Manager software (version 4.2) .

## Results

**Figure 1:**
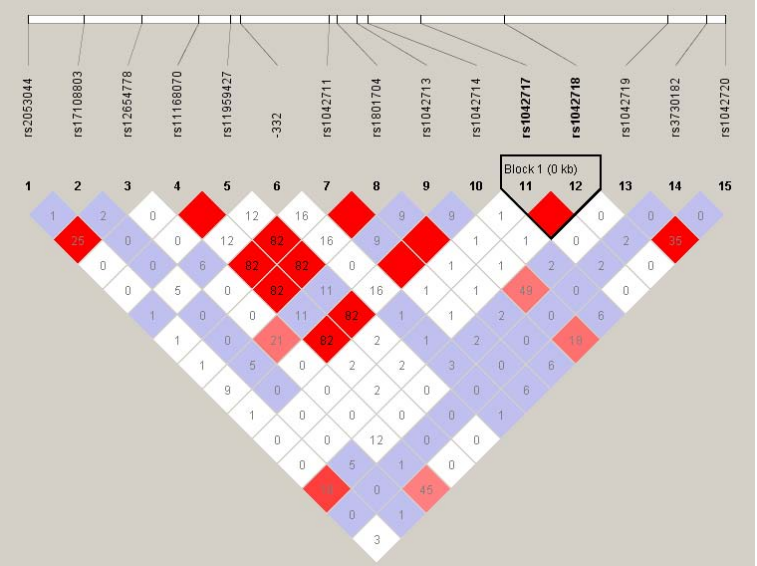
**Linkage disequilibrium (LD) plots of the fifteen SNPs in *ADRB2 *identified from 48 subjects**. We constructed the plots with the Haploview program [15], and r^2 ^(×100) values were depicted in the diamonds. Blocks were determined using the default method of Gabriel *et al *[16].

Fifteen polymorphisms were identified through the resequencing of *ADRB2 *for 48 individuals (Table 1), all of which were SNPs; one SNP (-332) located at position -332 upstream from the *ADRB2 *transcription initiation site was novel when compared with dbSNP Build 128. The nonsynonymous SNP rs1800888 at nt 491 (+491 C→T, 164 Thr→Ile) in the coding region was found not to be polymorphic in these 48 Chinese individuals. Among the fifteen SNPs, twelve SNPs had minor allele frequencies (MAF) great than 5% (Table 1), with frequencies ranging from 5.2% (rs1042711, rs1801704 and rs1042714) to 38.9% (rs1042719). The frequencies of the fifteen SNPs in 48 individuals were in Hardy-Weinberg equilibrium (P > 0.05, Table 1). The linkage disequilibrium (LD) structure among the SNPs was examined by program Haploview 4.0 (Figure 1). The LD between rs11959427 and rs1042714 (D' = 1, r^2 ^= 0.82) is similar to a previous report by Japanese researchers [6], but much stronger than that in the Polish study [7].

**Table 1:** Fifteen SNPs in the *ADRB2 *gene identified from 24 GD patients and 24 control subjects

Rs number	Position in *ADRB2*	Chromosome position	Alleles	HWpval	MAF
rs17108803	- 839	148185749	T:G	1.00	0.032
rs12654778	- 654	148185934	G:A	0.13	0.415
rs11168070	- 468	148186120	C:G	1.00	0.062
rs11959427	- 367	148186221	T:C	1.00	0.062
-332	- 332	148186256	G:A	1.00	0.042
rs1042711	- 47	148186541	T:C	1.00	0.052
rs1801704	- 20	148186568	T:C	1.00	0.052
rs1042713	+ 46	148186633	A:G	0.88	0.365
rs1042714	+ 79	148186666	C:G	1.00	0.052
rs1042717	+ 252	148186839	G:A	0.13	0.302
rs1042718	+ 523	148187110	C:A	0.13	0.302
rs1042719	+ 1053	148187640	G:C	0.60	0.389
rs3730182	+ 1179	148187766	T:C	1.00	0.011
rs1042720	+ 1239	148187826	A:G	0.58	0.352

SNP positions in the *ADRB2 *gene were numbered upstream (+) or downstream (-) from the *ADRB2 *transcription initiation site. Chromosome positions were referred to the sequence of NCBI database (built 35). SNP, single nucleotide polymorphism; Hwpval, Hardy-Weinberg equilibrium p value; MAF, minor allele frequency.

All the fifteen SNPs were subsequently included in the tag SNP selection, and ten tag SNPs were selected and genotyped in the case-control collection. Allele and genotype frequencies for these tag SNPs are shown in Table 2. All tag SNPs genotypes in cases and controls conformed to Hardy-Weinberg equilibrium (P > 0.001). Genotype distributions and allele frequencies of the ten tag SNPs in *ADRB2 *were not significantly different between the GD group and the control group (Table 2). When under the assumption of dominant and recessive model, all the tag SNPs failed to show associations with GD either (data not shown here). Since the association of rs1042714 with GD was stronger in the patients without symptoms of GO in the Polish study[7], we also compared the allele and genotype frequencies of rs1042714 in GD subjects with or without GO to controls, and observed no difference existed (Table 3). No blocks were detected based on the ten tag SNPs. To explore the associations of haplotype rs11959427/rs1042713/rs1042714 (-367T/+46A/+79C) with GD as previously reported[7], we inferred the haplotypes based on rs1042713 and rs1042714, since rs11959427 captured by rs1042714 at r^2 ^≥ 0.8 were not directly genotyped in our case-control collection. Three common haplotypes were observed and none of them showed statistically significant association with GD (Table 4).

**Table 2:** Association analysis of the ten tag SNPs in GD patients and control subjects

	Allele	Genotype of case			Genotype of control			Allele comparison		Genotype comparison
	
Rs number	1/2	11	12	22	11	12	22	OR (95% CI)	P value	P value
rs2053044	G/A	220	167	32	375	251	52	0.932(0.768–1.131)	0.48	0.63
rs17108803	T/G	390	33	2	596	74	4	1.420(0.960–2.120)	0.08	0.21
rs12654778	G/A	161	196	68	254	316	112	1.022(0.858–1.219)	0.80	0.97
-332	G/A	410	15	1	645	28	0	1.043(0.568–1.918)	0.89	0.41
rs1042713	A/G	170	179	76	248	301	123	1.076(0.903–1.283)	0.41	0.58
rs1042714	C/G	358	63	3	592	88	5	0.870(0.631–1.199)	0.39	0.64
rs1042717	A/G	199	168	45	312	276	83	1.077(0.894–1.298)	0.43	0.73
rs1042719	G/C	137	216	66	213	318	139	1.128(0.947–1.343)	0.18	0.11
rs3730182	T/C	401	18	0	647	22	1	0.831(0.448–1.540)	0.56	0.64
rs1042720	A/G	155	208	56	280	293	94	0.913(0.763–1.091)	0.32	0.17

**Table 3:** Association analysis of the rs1042714 polymorphism in control subjects and GD patients with or without GO.

	Genotype			Allele comparison		Genotype comparison
			
	CC	CG	GG	OR (95% CI)	P value	P value
Control	592	88	5			
GO (-)	248	45	2	1.18(0.82–1.68)	0.38	0.60
GO (+)	110	18	1	1.09(0.66–1.80)	0.73	0.94

**Table 4:** Estimated frequencies of the haplotypes inferred from rs1042713 and rs1042714 in GD patients and controls

Haplotype	Frequency (%)		P value
		
rs1042713, rs1042714	Case	Control	
AC	0.584	0.584	0.996
GC	0.336	0.344	0.682
GG	0.080	0.072	0.482

**Figure 2:**
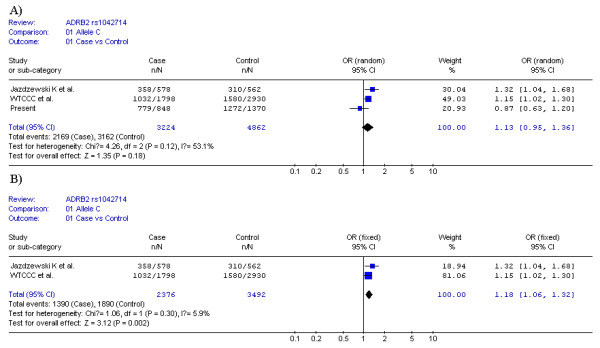
**Meta-analysis of the association between rs1042714 and GD measured by allele frequency data**. OR (black squares) and 95% CI (bar) are shown for each study. The pooled ORs and their 95% CIs are represented by the shaded diamonds. Summary ORs are given for each ethnic group as well as all groups combined. The symbol n indicates the total number of C alleles, and N indicates the total number of C plus G alleles. A) DerSimonian-Laird OR meta-analysis (random effects) of the three studies; B) Mantel-Haenszel OR meta-analysis (fixed effects) of the two European studies.

A meta-analysis for rs1042714 was conducted combining the initial findings from the Polish populations, the data set from the study of WTCCC et al. and the result of present study, including a total of 1,728 GD patients and 2,491 control samples. The forest plots for the allele model are shown in Figure 2. The p-value for Q-test is just above the significance level (P = 0.12, Figure 2A), whereas the I^2 ^statistic suggested there was significant heterogeneity among the studies (I^2 ^= 53.1%, Figure 2A). Since only three studies were included in our meta-analysis, we chose the result of I^2 ^statistic as measurement of heterogeneity. Then the pooled OR was calculated by random effects approaches using DerSimonian and Laird methods[20]. The pooled OR was 1.13 but the confidence interval spanned unity (95% CI, 0.95 to 1.36), which indicated the combined data did not show significant association for rs1042714 with GD. A sensitivity analysis showed that the present study in Chinese population was the main cause of the heterogeneity. When only the two European studies (including 1300 cases and 1801 controls) were combined, the heterogeneity was no longer significant (I^2 ^= 5.9%, Figure 2B), and the result demonstrated significant association by fixed effect (OR = 1.18, 95% CI, 1.06 to 1.32; P = 0.002, Figure 2B).

## Discussion

We have performed a comprehensive study investigating association of GD susceptibility with the *ADRB2 *variants and failed to replicate association in Chinese population. However, meta-analysis revealed that rs1042714 was significantly associated with GD in Caucasian population. Genetic association studies of GD applied to other non-*MHC *candidate genes have also led to encouraging yet somewhat inconsistent findings in recent years. Several reasons may explain these inconsistent findings, including allelic heterogeneity, true variation in disease association between populations, modifying genetic and/or environmental factors, statistically underpowered small sample sizes, and a low prior probability of obtaining a true result[21]. The most parsimonious explanation for the lack of replication of initial *ADRB2 *association results in our study would be variation in disease association between populations and relatively small sample size.

It has long been noted that the MAF of rs1042714 locus varies significantly among the major populations around the world[5,22]. The minor allele of rs1042714 loci is much more common in Caucasians than in Africans and Asians according to the previous reports and Hapmap data[5,22]. Xie *et al *[22]and Chang *et al *[23] observed that the MAF of rs1042714 locus was 7.2% among 104 healthy Chinese and 7.7% among 260 nondiabetic Chinese respectively. The Hapmap data revealed the MAF of rs1042714 locus was 12.2% among 45 Chinese. In our study, the MAF of rs1042714 locus among the 690 healthy controls was 7.2%, consistent with the previous report. In the previous reports of Jazdzewski K *et al *in Polish population[7] and WTCCC *et al *in UK population[8], carriers of the minor allele G were present less frequently in the patient groups (38% and 43% respectively) than in the control groups (45% and 46% respectively), whereas in our data of Chinese population, the proportion of minor allele G carriers was larger in cases (8%) than in controls (7%). The trend of rs1042714 allele effect in cases and controls was substantially different in the two populations, which was also reflected by the OR value in the meta-analysis (less or greater than 1, Figure 2A). The allelic heterogeneity and opposite effect directions might indicate variations in disease associations between populations.

Genetic heterogeneity across populations had previously been reported in GD and other polygenic autoimmune diseases, such as in the case of *SUMO4 *gene association with type 1 diabetes, GD, or rheumatoid arthritis (RA) [24-33]. *SUMO4 *gene was suggested as a general autoimmunity locus in the Asian population, associations with type 1 diabetes, GD and RA have been reported[27,31-33]. In contrast to the consistent association observed in the Asian populations, no evidence of *SUMO4 *association with susceptibility to type 1 diabetes, GD and RA was found from several studies in European descent[26,28-30]. One study did show SUMO4 was associated with T1D in association with high risk HLADR3 and DR4 genotypes in Swedish patients. But SUMO4 by itself was not associated with the disease[34]. Interestingly, a tendency for an opposite association was reported in type 1 diabetes of British subjects[25].

The major limitation of our case-control study was that the sample size was not very compelling. Sample size in thousands of subjects might be more sufficient to detect small genetic effects. However, positive association was reported from analysis of 300 GD patients and 301 control samples in Polish population, and positive signal was also found from the GWA screen of 1,000 GD patients and 1,500 control samples in UK population[7,8]. This prompted us to investigate the potential association between *ADRB2 *and GD in fair number of well-defined cases and controls (428 GD cases and 690 controls) in Chinese population. Assuming a frequency of the risk allele of 0.075, our study in 1,118 subjects had only 70% power to detect the OR of 1.5 for rs1042714 with a significance level of 0.05. We sought to increase the effective sample size and statistical power by meta-analysis and to draw a more compelling result, but heterogeneity was found to exist between European studies and our study, and the data involved in *ADRB2 *association with GD in Chinese and Caucasian populations must be analyzed separately. The possibility that SNPs in *ADRB2 *may influence the disease risk modestly in Chinese population cannot be excluded completely. Further accumulation of samples would be needed for ascertainment. The overall sample size included in the meta-analysis was relative large (3101 Caucasian individuals) and this meta-analysis provided an up-to-date summary of the association analysis between *ADRB2 *and GD. However, the number of published studies available for meta-analysis was small, which made our analysis more prone to publication bias. Thus, it seems a little premature to draw conclusions that *ADRB2 *is associated with GD susceptibility in Caucasian populations.

A GD linkage locus had been mapped to chromosome 5q31-33 in Asian population in two GD genome screen studies [2,4], but not detected in a Caucasian population[35]. The genome wide association study in Caucasians revealed some positive signal in this region[8], albeit not as strong as other loci. Our meta-analysis showed association of the *ADRB2 *variation with GD in Caucasians, which provided new evidence for the presence of GD susceptibility genes in 5q31-33. Much effort has been spent in exploring the associated genes and/or the causal allele(s) of Crohn disease[36,37] and rheumatoid arthritis[38] located on the chromosome region of 5q31-33, and great progress has been made. However, no confirmed GD association genes have been identified in this region so far. Of note, different autoimmune diseases may share similar genetic etiologies and susceptibility alleles; therefore, the data sets from Crohn disease and rheumatoid arthritis studies will be helpful in further clarifying the variant accounted for susceptibility to GD in this region. A more comprehensive analysis of chromosome 5q31-33, including a wider range of SNPs in *ADRB2 *and neighboring genes in a large, well matched dataset, is required to determine whether a causal variant within any candidates in this linkage region confers susceptibility to GD.

## Conclusion

We systematically studied the association of the *ADRB2 *gene by a case-control analysis using tag SNPs and a meta-analysis. The case-control study did not support *ADRB2 *as a GD susceptibility gene in Chinese population, but our meta-analysis provided strong evidence of a positive association of the *ADRB2 *SNP rs1042714 with GD in Caucasians. More studies with large sample size in Chinese and other populations are needed before solid conclusions about the association could be made.

## Competing interests

The authors declare that they have no competing interests.

## Authors' contributions

XC designed the study, carried out the statistical analysis, and wrote the manuscript. MS, BW, KZ and QH conducted the molecular genotyping of the variants. LS and YD were involved in the recruitment of the patients and controls. CD, YW and SX assisted in manuscript revising. HW conceptualized the study, supervised the study and obtained the funding. All authors read and approved the final manuscript.

## Pre-publication history

The pre-publication history for this paper can be accessed here:


